# Synchronous Firefly Algorithm for Cluster Head Selection in WSN

**DOI:** 10.1155/2015/780879

**Published:** 2015-09-30

**Authors:** Madhusudhanan Baskaran, Chitra Sadagopan

**Affiliations:** ^1^Department of Computer Science, Er.Perumal Manimekalai College of Engineering, Hosur, Tamil Nadu 635117, India; ^2^Er.Perumal Manimekalai College of Engineering, Hosur, Tamil Nadu 635117, India

## Abstract

Wireless Sensor Network (WSN) consists of small low-cost, low-power multifunctional nodes interconnected to efficiently aggregate and transmit data to sink. Cluster-based approaches use some nodes as Cluster Heads (CHs) and organize WSNs efficiently for aggregation of data and energy saving. A CH conveys information gathered by cluster nodes and aggregates/compresses data before transmitting it to a sink. However, this additional responsibility of the node results in a higher energy drain leading to uneven network degradation. Low Energy Adaptive Clustering Hierarchy (LEACH) offsets this by probabilistically rotating cluster heads role among nodes with energy above a set threshold. CH selection in WSN is NP-Hard as optimal data aggregation with efficient energy savings cannot be solved in polynomial time. In this work, a modified firefly heuristic, synchronous firefly algorithm, is proposed to improve the network performance. Extensive simulation shows the proposed technique to perform well compared to LEACH and energy-efficient hierarchical clustering. Simulations show the effectiveness of the proposed method in decreasing the packet loss ratio by an average of 9.63% and improving the energy efficiency of the network when compared to LEACH and EEHC.

## 1. Introduction

**Figure 1:**
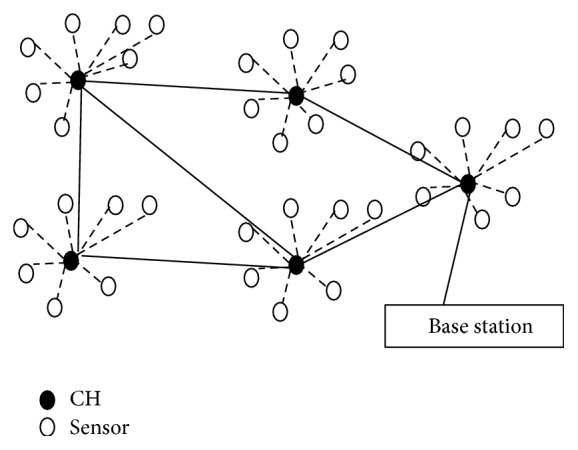
WSN architecture.

Wireless Sensor Network (WSN) finds extensive application in both civilian and military applications. It has been extensively used in target tracking, surveillance, monitor natural disasters, biomedical applications, habitat monitoring, and building management systems [1]. Sensor nodes in natural disasters sense/detect an environment to forecast disasters. In biomedical applications, sensor surgical implants monitor patient's health. In seismic sensing, sensors ad hoc deployment in a volcanic area detects earthquakes/eruptions [2]. WSN nodes use nonrechargeable storage device with restricted energy and in general replacing batteries is not possible. Thus, energy efficiency is an important issue, and designing power-efficient protocols is critical to prolong life of the sensors [3]. Typically WSNs monitor specific areas using sensors collect data and send to base station (BS). A typical WSN organized hierarchically is shown in Figure 1. In hierarchical structure, to save energy some nodes selected based on the objective function act as Cluster Head (CH) and aggregate data from its entire neighbor. The CH then sends the data to the BS and thus reduces network overheads to ultimately save energy in each node.

WSNs unlike traditional networks have their own design/resource constraints which include limited energy, shorter transmission range, limited bandwidth, and minimal processing power in nodes. Based on the deployment scheme, network size varies with the environment. One of the most important activities in WSN is data aggregation which is the process of gathering data from multiple sensors, fusion of the data, and reduction of redundant transmission. Hierarchical techniques have been found to be quite effective in data aggregation.

LEACH randomizes rotation of nodes as CH and thus distributes energy load among network sensors evenly. The idea of LEACH protocol is that nodes become CH periodically with every period having two stages. The first stage is cluster construction and the next is data communication [4]. During the cluster formation each node selects a random number and compares with threshold values *t*(*n*). If the number is less than *t*(*n*), then it is chosen as CH; else it remains as a regular node in that round. The threshold *t*(*n*) is given by

(1) $$\begin{array}{c} t\left(\;n\;\right)=\left\{\begin{array}{cc} \frac{p}{1-p*\left(r\mathrm{mod}\left(1/p\right)\right)} & \text{if }n\in G\\ 0 & \text{if }n\notin G, \end{array}\right. \end{array}$$

where *p* is the percentage of the Cluster Heads over all nodes. *r* is the round number. *G* is the set of nodes that have not been CH in the first 1/*p* rounds.

Table 1 reviews popular hierarchical data aggregation protocols [5].

From Table 1 it can be observed that clustering objectives are varied with one of the key objectives being improvement in the network lifetime. Often a clustering objective facilitates meeting application requirements [6–10].

As LEACH depends on probability model efficiency in energy savings may not be obtained as CHs may be close to each other [11]. To overcome the disadvantages of LEACH many protocols have been proposed in literature to overcome the suboptimal solution. Various heuristic algorithms based on Genetic Algorithm (GA), Particle Swarm Optimization (PSO), and Artificial Bee Colony (ABC) algorithm have been proposed.

In this work, investigations were carried out using the firefly heuristic. A novel firefly heuristic to avoid the local minimum problem is proposed. Firefly heuristic is based on the light intensity produced by fireflies. The intensity of light produced is mapped to the objective function and hence fireflies with low intensity are attracted towards fireflies with higher light intensity. In this work, a hybrid firefly algorithm, synchronous firefly algorithm, is proposed based on

- ranked sexual reproduction capability of select fireflies,
- the fireflies created by this method having the best genes from the ranked fireflies.

The advantages of the proposed technique are

- faster convergence,
- avoidance of multiple local optima.

## 2. Related Work

Hussain et al. [12] presented a wide taxonomy on CH selection techniques in WSN with comparative analysis. Hu et al. [13] proposed a multihop heterogeneous cluster-based optimization algorithm (MHCOA) which reduces the number of CHs, saving an average of 16.7% network energy with minimal end to end delay. Peng et al. [14] presented an energy-efficient prediction clustering algorithm to reduce energy consumption when broadcasting in clustering phase and prolong network lifetime. Bencan et al. [15] proposed an energy-heterogeneous clustering scheme (EHCS) which allows variations in the initial energy of the nodes based on the distance to sink to avoid the energy-hole problem.

Optimization techniques for cluster formation and CH selection using PSO, GA, and ACO have been extensively proposed in the literature. Kuila and Jana [16] proposed PSO based CH selection technique with a multiobjective function considering energy consumption of the CHs and delay in forwarding the data packets. In the proposed system, each particle's dimension is equal to the number of sensor nodes in the network. Natarajan et al. [17] applied LEACH and PSO for optimal selection of energy-aware clusters and CHs. Ma et al. [18] proposed Dual Cluster Heads using Niching Particle Swarm Optimization (DCH-NPSO) which generated two CHs per cluster: Master Cluster Head (MCH) and the Slave Cluster Head (SCH). Ma et al. [19] proposed Adaptive Assistant-Aided Clustering Protocol using Niching Particle Swarm Optimization (AAAC-NPSO) to improve system lifespan and data delivery by optimizing energy dissipation in the networks. Shankar et al. [20] discussed an optimal radius algorithm and hybrid PSO algorithm for selection of CH to extend the lifetime of the network. Ali and Shahzad [21] studied PSO, ACO for routing overhead, route optimality, and energy consumption. Simulation results conclude that PSO and ACO based protocols are efficient routing optimization approaches for WSNs.

Rana and Zaveri [22] proposed an integrated approach of CH selection and routing in two-tier WSN using GA based CH selection with A-Star algorithm based routing to extend life of WSN. This approach leads to significant improvements in the network lifetime over other techniques. Peiravi et al. [23] proposed a multiobjective two-nested Genetic Algorithm (GA) based clustering for optimizing the network lifetime for different delay values. Kuila et al. [24] proposed a GA based load balanced clustering algorithm for WSN. Gupta et al. [25] presented GA based routing (GAR) to optimize the distance travelled by the data to reduce energy consumption. New routing schedules were calculated by the proposed GA based on the current network state. Cheng et al. [26] presented Dynamic Load Balanced Clustering Problem (DLBCP) and a dynamic GAs based solution to solve the dynamic network optimization requirements. Özdemir et al. [27] employed Multiobjective Evolutionary Algorithm based on Decomposition (MOEA/D) to optimize cluster-based WSNs. The proposed technique improved coverage and network lifetime compared to NSGA II.

Karaboga et al. [28] presented a novel energy-efficient clustering mechanism, based on Artificial Bee Colony algorithm to prolong the network lifetime. Kumar and Kim [29] proposed a new Efficient Learning Automata Based Cell Clustering Algorithm (ELACCA) for WSNs. Hoang et al. [30] proposed a harmony search algorithm for development of centralized cluster-based protocols by minimizing the intracluster distances between the cluster members and their CHs.

From literature it can be seen that GA, PSO, and ACO have been extensively used for CH selection. Though GA has good global search characteristics, convergence is poor. Representation of weights in PSO is done arbitrarily and hence search is limited to either global or local space. In this work, it is proposed to investigate the firefly metaheuristic which finds optimal global solution with fast convergence even under multiparameter optimization strategy.

Yang [31, 32] demonstrated that though PSO achieves better global optima for various test functions for multimodal optimization than GA firefly algorithm is superior to both PSO and GA in terms of both efficiency and success rate. Similarly, Łukasik and Żak [33] demonstrated the superiority of firefly algorithm in continuous constrained optimization tasks when compared to PSO. Fister Jr. et al. [34] reviewed the use of firefly algorithm in various application domains. The authors conclude that the firefly can efficiently handle multimodal problems, has fast convergence, and is effectively used for general, global, and also local search heuristic.

## 3. Problem Formulation

WSN can be represented by graph *G* = (*V*, *E*) with vertices *V* = {*v*_1_, *v*_2_,…, *v*_*n*_} and edges *E* = {*e*_1_, *e*_2_,…, *e*_*m*_}. Each edge has weights which represents certain network parameters denoted by

(2) $$\begin{array}{c} w_{i}=\left(\;w_{1i},w_{2i},w_{3i},\ldots ,w_{pi}\;\right)\;i=1\text{,}\ldots ,m, \end{array}$$

where *w*_*ki*_ (*k* = 1,2,…, *p*) represents parameters of the network.

*x* = (*x*_1_, *x*_2_ …, *x*_*m*_) can be defined by

(3) $$\begin{array}{c} x_{i}=\left\{\begin{array}{cc} 1 & \text{if }e_{i}\text{ selected}\\ 0 & \text{otherwise}. \end{array}\right. \end{array}$$

The objective of the heuristic algorithm is given by

(4) $$\begin{array}{c} \min z_{1}\left(\;x\;\right)=\overset{m}{\underset{i=1}{\sum }}w_{1i}x_{i}\\ \min z_{2}\left(\;x\;\right)=\overset{m}{\underset{i=1}{\sum }}w_{2i}x_{i}\\ \vdots \\ \min z_{p}\left(\;x\;\right)=\overset{m}{\underset{i=1}{\sum }}w_{pi}x_{i}, \end{array}$$

where *z*_*i*_(*x*) is the *i*th objective to be minimized for the problem.

In this work, three quality of service parameters, packet loss rate, end to end delay, and remaining energy, are considered to build the objective function as a minimization problem.

## 4. Methodology

**Figure 2:**
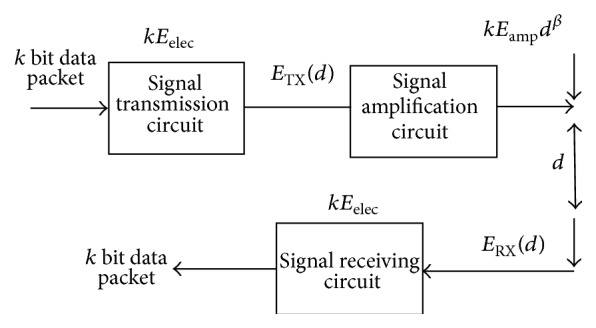
The first-order energy model.

The first order energy model for energy consumed when communication occurs between two nodes is shown in Figure 2.

For distance *d* between two nodes, the transmitter energy consumption [34] for transmitting *k* bit is given by

(5) $$\begin{array}{c} E_{\text{TX}}\left(\;k,d\;\right)=\left\{\begin{array}{cc} kE_{\text{elec}}+k\varepsilon_{\text{fs}}d^{2}, & d<d_{0}\\ kE_{\text{elec}}+k\varepsilon_{\text{amp}}d^{4}, & d>d_{0}. \end{array}\right. \end{array}$$

The energy consumed by the receiver is given by

(6) $$\begin{array}{c} E_{\text{RX}}\left(\;k\;\right)=kE_{\text{elec}}. \end{array}$$

In the above equations, transmitting and receiving 1 bit data's energy consumption is denoted by *E*_elec_. *ε*_fs_, *ε*_amp_ represent the coefficients of energy consumption for different channel propagation models. *d*_0_ is a threshold value denoted as $d_{0}=\sqrt{\varepsilon_{\text{fs}}/\varepsilon_{amp}}$, to distinguish free-space path loss model from a multipath fading model. Energy consumption for integration of *l* data packets of *k* bit is expressed as *E*_DA_(*k*) = *l∗k∗E*_DA_, where *E*_DA_ is energy consumption for integration of data of 1 bit.

### 4.1. Proposed Firefly for Cluster Head Formation

Firefly algorithm metaheuristics work on the principle of the flashing lights of fireflies. The intensity of the light helps a firefly swarm move to brighter and attractive locations which can be mapped to an optimal solution in the search space. The algorithm standardizes some of the firefly characteristics and can be listed as follows:

- Each firefly can be attracted to another irrespective of their sex.
- The brightness produced by the firefly is directly proportional to its attractiveness and between two fireflies, the firefly with higher brightness attracts the one which has lower brightness. A firefly moves randomly if it is not able to find a brighter neighboring firefly.
- In the mathematical model, firefly's brightness is based on the objective function.

Firefly metaheuristic is chosen for its capability of providing optimal solutions for multiobjective problems. In this work, a novel fitness function considering energy, end to end delay, and packet loss rate is proposed and given by

(7) $$\begin{array}{c} F\left(\;x\;\right)=\frac{\left(P_{d}/P_{t}\right)\times \left(E_{i}^{r}/E_{\text{init}}\right)}{\exp^{-e_{d}/e_{m}}}, \end{array}$$

where *P*_*d*_ is the number of dropped packets. *P*_*t*_ is the total number of packets sent. *E*_*i*_^*r*^ is the remaining energy in node *i*. *E*_init_ is the initial energy. *e*_*d*_ is the end to end delay. *e*_*m*_ is the maximum allowable delay.

The cluster formation and CH selection in firefly are given in Algorithm 1.

In firefly algorithm [30], variation of light intensity and the formulation of the problem in terms of attractiveness are crucial as the objective function is encoded into it. The light intensity is calculated using *γ*; the fixed light absorption coefficient and the light intensity *I* can be computed based on distance *r* such that

(8) $$\begin{array}{c} I=I_{0}e^{-\gamma r}, \end{array}$$

where *I*_0_ is the original light intensity. Approximating using Gaussian law we have

(9) $$\begin{array}{c} I\left(\;r\;\right)=I_{0}e^{-\gamma r^{2}}. \end{array}$$

The attractiveness *β* of a firefly is given in

(10) $$\begin{array}{c} \beta \left(\;r\;\right)=\beta_{0}e^{-\gamma r^{2}}, \end{array}$$

where *β*_0_ is the attractiveness at *r* = 0.

In two-dimensional space the distance between two fireflies can be given by their Euclidean distance as $r_{ij}=\sqrt{{\left(x_{i}-x_{j}\right)}^{2}+{\left(y_{i}-y_{j}\right)}^{2}}$. A firefly *i* moves to a more attractive firefly *j* by

(11) $$\begin{array}{c} x_{i}=x_{i}+\beta_{0}e^{-\gamma r^{2}}\left(\;x_{j}-x_{i}\;\right)+\alpha \left(\;\text{rand}-\frac{1}{2}\;\right). \end{array}$$

In this work, binary values are used to represent the nodes in each solution. The challenge in this type of encoding is between the real-valued vector space *ℜ* ^*N*^ and binary space {0,1}^*N*^ and given by

(12) $$\begin{array}{c} X_{ik}=\left\{\begin{array}{cc} 1, & \text{if }\mathrm{rand}\left(\right)\leq \frac{1}{1+\exp\left(-X_{ik}\right)}\\ 0, & \text{otherwise}, \end{array}\right. \end{array}$$

where *k* = 1,…, *N* and rand⁡() ~ *U*(0,1).

In the proposed synchronous firefly algorithm, the fireflies are ranked and the best fireflies selected using tournament selection. The selected fireflies reproduce among themselves by crossover and mutation. An example of the proposed technique is shown.  Table 2 shows the partial solution for the best fireflies obtained using tournament selection.

After crossover and mutation, the reproduced fireflies are given by Table 3.

The new solutions are added to the firefly pool and the next iteration of the firefly is continued.

### 4.2. Parameters for Network Simulation

The performance evaluation of the proposed algorithm was carried out using MATLAB. The base station is located 50 meters away from (0,0) of the network. The base station is assumed to have infinite power source:

- Nodes are static and do not change location after deployment.
- All nodes have uniform energy at the time of deployment.
- Base station is located outside the network area.
- Each node has a unique ID.
- The transmission power in the node varies based on the distance between the communicating devices.

Table 4 shows the simulation parameters used in this work.

## 5. Result and Discussion

**Figure 3:**
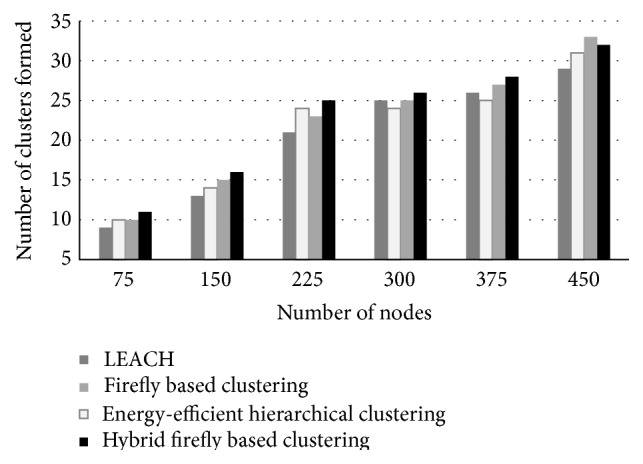
Number of clusters formed.

**Figure 4:**
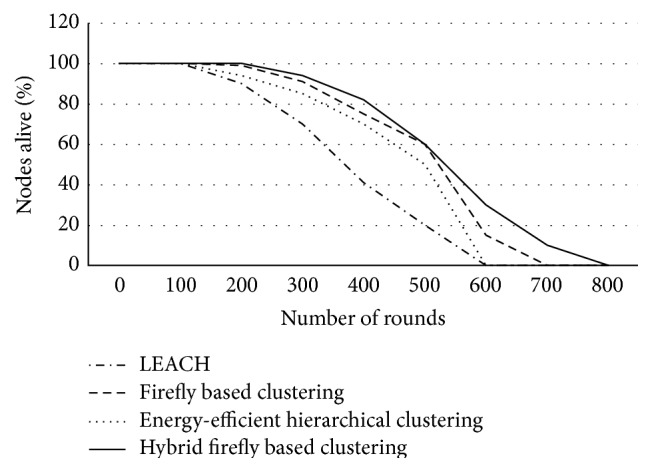
Lifetime computation.

**Figure 5:**
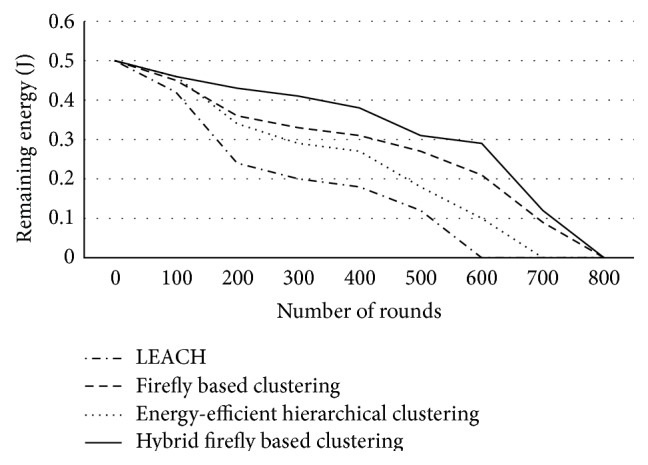
Remaining energy computation.

Simulations were carried out using LEACH, EEHC, firefly, and synchronous firefly algorithm. LEACH was used to compare the proposed algorithm due to its popularity in the literature and being a random method.  Table 5 tabulates the simulation results of packet loss rate and end to end delay for various clustering techniques. Figures 3–5 show the results number of clusters formed, lifetime computation, and remaining energy, respectively.

The proposed hybrid firefly algorithm minimized the packet loss rate by 2.27% when compared to firefly based clustering with 225 nodes and by 39.74% when compared to LEACH with 450 nodes. The proposed hybrid firefly algorithm minimized the end to end delay by 6.42% when compared to firefly based clustering with 450 nodes and by 8.69% when compared to LEACH with 150 nodes.  Figure 3 shows number of clusters formed for various clustering techniques for different number of nodes.

It is observed that the proposed algorithm increases the number of clusters hence reducing the energy consumption significantly.  Figure 4 shows the lifetime computation in the form of percentage of nodes alive for various clustering techniques when the number of nodes used is 225.

The proposed hybrid firefly algorithm increased lifetime by 66.67% when compared to firefly based clustering in 400 rounds and by 66.67% when compared to LEACH in 600 rounds.

Figure 5 shows the remaining energy computation for various clustering techniques when the number of nodes is 225. The proposed hybrid firefly algorithm has an overall average remaining energy of 88.37% when compared to firefly based clustering in 500 rounds and by 28.57% when compared to LEACH in 600 rounds.

## 6. Conclusion

This work proposed a novel firefly based clustering protocol to select Cluster Head in WSNs. LEACH protocol needs the user to specify probability for use with a threshold function to determine whether a node will become a CH or not leading to NP problem. In the proposed hybrid firefly algorithm, the best fireflies selected using tournament selection are allowed to reproduce among themselves by crossover and mutation. The proposed method achieves faster convergence and avoids multiple local optima. Simulation results demonstrate the efficiency of the proposed method in decreasing the packet loss rate by 15.4% to 39.74% when compared to LEACH and by 6.16% to 30.66% when compared to energy-efficient hierarchical clustering. The proposed hybrid firefly algorithm also increased the lifetime of the network. Future work can be carried out to investigate the impact on increasing specific quality of service parameter.

## Figures and Tables

**Algorithm 1:**
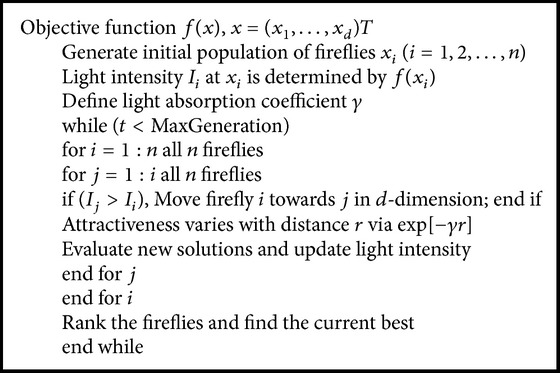
Pseudocode for cluster formation and CH selection in firefly.

**Table 1:** Information maintained in the neighborhood table.

Protocol	Organization type	Objectives	Characteristics
LEACH	Cluster	Improve network life time	CHs are rotated randomly for specific time using threshold.

HEED	Cluster	Increase number of rounds	Nodes with different power levels are assumed.

PEGASIS	Chain	Average energy spent by node	Network knowledge is required for computation

Hierarchical chain based protocols	Chain	Energy × delay	Uses chain scheme with binary values.

EADAT	Tree	Improves the number of available nodes at each round.	Broadcasting is achieved from sink

PEDAP-PA	Tree	Balances node dissipation such that all nodes die simultaneously.	Uses the popular Minimum Spanning Tree to achieve its goal

**Table 2:** 

Firefly	n1	n2	n3	n4	n5	n6	n7	n8	n9	n10

soln 1	0	1	0	0	1	1	1	1	1	1

soln 2	0	1	1	0	1	1	0	1	0	0

soln 3	0	0	1	1	0	1	0	1	1	0

**Table 3:** 

newsoln 1	0	1	0	0	0	1	0	1	0	0

newsoln 2	0	1	1	0	1	0	1	0	1	1

newsoln 3	0	1	0	0	1	1	0	0	1	0

newsoln 4	0	0	1	0	0	1	1	0	1	1

**Table 4:** Simulation parameters.

Parameters	Values
Initial energy of nodes *E*_init_	0.5 J
Amplification coefficient of the free space model *E*_fs_	10 pJ·m^2^/b
Amplification coefficient of the multipath transmission model *E*_amp_	0.0013 pJ·m^2^/b
Table data fusion rate *E*_DA_	5 nJ/b
Circuit loss *E*_elec_	50 nJ/b
Clustering probability of nodes *p*	0.05
Data packet length	4000 b
Control packet length	80 b

**Table 5:** Average packet loss rate and end to end delay.

Number of nodes	LEACH	Energy efficient hierarchical clustering	Firefly based clustering	Hybrid firefly based clustering
Packet loss ratio %				
75	8.3	7.52	6.93	6.07
150	12.74	11.34	10.2	9.06
225	13.15	12.66	10.66	10.42
300	18.3	17.06	15.62	15.53
375	24.68	23.04	21.26	21.05
450	34.48	32.76	24.08	23.05

End to end delay in second				
75	0.0011	0.0012	0.0012	0.0010
150	0.0012	0.0014	0.0013	0.0011
225	0.0116	0.0131	0.0121	0.0109
300	0.0197	0.0160	0.0146	0.0136
375	0.0404	0.0421	0.0369	0.0352
450	0.0436	0.0440	0.0466	0.0437
